# Amelogenin-Derived Peptide (ADP-5) Hydrogel for Periodontal Regeneration: An In Vitro Study on Periodontal Cells Cytocompatibility, Remineralization and Inflammatory Profile

**DOI:** 10.3390/jfb14020053

**Published:** 2023-01-18

**Authors:** Nina Attik, Xavier Garric, Audrey Bethry, Gilles Subra, Charlène Chevalier, Brahim Bouzouma, Pascal Verdié, Brigitte Grosgogeat, Kerstin Gritsch

**Affiliations:** 1 Laboratoire des Multimatériaux et Interfaces, UMR CNRS 5615, Université Claude Bernard Lyon 1, Université de Lyon, 69622 Villeurbanne, France; 2 Faculté d’Odontologie, Université Claude Bernard Lyon 1, Université de Lyon, 69008 Lyon, France; 3 Institut des Biomolécules Max Mousseron (IBMM), University of Montpellier, CNRS, ENSCM, 34000 Montpellier, France; 4 Departement of Pharmacy, Nîmes University Hospital, 30900 Nîmes, France; 5 Service d’Odontologie (UF Recherche Clinique), Hospices Civils de Lyon, 69007 Lyon, France; 6 Service d’Odontologie (UF Parodontologie), Hospices Civils de Lyon, 69007 Lyon, France

**Keywords:** amelogenin-derived peptide, hydrogel, biocompatibility, bioactivity, inflammatory mediators, periodontal cells, mineralization, periodontal regeneration, scaffold

## Abstract

A relevant alternative to enamel matrix derivatives from animal origin could be the use of synthetic amelogenin-derived peptides. This study aimed to assess the effect of a synthetic amelogenin-derived peptide (ADP-5), alone or included in an experimental gellan–xanthan hydrogel, on periodontal cell behavior (gingival fibroblasts, periodontal ligament cells, osteoblasts and cementoblasts). The effect of ADP-5 (50, 100, and 200 µg/mL) on cell metabolic activity was examined using Alamar blue assay, and cell morphology was assessed by confocal imaging. An experimental gellan–xanthan hydrogel was then designed as carrier for ADP-5 and compared to the commercial gel Emdogain^®^. Alizarin Red was used to determine the periodontal ligament and cementoblasts cell mineralization. The inflammatory profile of these two cells was also quantified using ELISA (vascular endothelial growth factor A, tumor necrosis factor α, and interleukin 11) mediators. ADP-5 enhanced cell proliferation and remineralization; the 100 µg/mL concentration was more efficient than 50 and 200 µg/mL. The ADP-5 experimental hydrogel exhibited equivalent good biological behavior compared to Emdogain^®^ in terms of cell colonization, mineralization, and inflammatory profile. These findings revealed relevant insights regarding the ADP-5 biological behavior. From a clinical perspective, these outcomes could instigate the development of novel functionalized scaffold for periodontal regeneration.

## 1. Introduction

Periodontal disease is a highly prevalent infectious disease that induces inflammation of periodontal tissues until their destruction [1]. Periodontal inflammation also has systemic consequences that can aggravate certain pathologies such as diabetes, cardiovascular diseases, and rheumatoid arthritis [2,3]. Moreover, it could be correlated with pregnancy complications such as underweight and preterm infants [4,5]. Periodontal therapy can successfully stop the disease progression by targeting the microbial etiology of the disease; this allows the recovery of periodontal health and also can improve the conditions of certain pathologies associated with periodontitis [6,7]. However, one of the remaining challenges is the regeneration of the destroyed tissues, i.e., new cementum, periodontal ligament, and bone coronal to the base of the defect [8]. This regeneration through the regaining of the initial volume and quality of the periodontal tissues reduces the risk of tooth loss and masticatory dysfunction in case of recurrence of the periodontitis [9]. Many regenerative techniques and biomaterials have been developed and evaluated in vitro, preclinical, and/or clinical studies. Nevertheless, despite the clinically positive results, periodontal regeneration remains incomplete and unpredictable, particularly in advanced defects [8,10,11].

Hence, there is a need for new molecules of interest that could be easily produced and integrated into different types of scaffolds such as hydrogels and nanofibers [12]. Iviglia et al. [13], in their review, suggested functionally graded scaffolds as a valuable approach with an enhanced potential to manage periodontal lesions. Therefore, the application of these shapeable and functionalized scaffolds could be able to fit the periodontal lesions’ morphological features and take into account the periodontal tissues’ plurality regeneration time. Currently, the Emdogain^®^ gel (Straumann, Switzerland), containing enamel matrix derivatives (EMDs) and extracted from developing porcine teeth is commercially available. There is ample evidence of its value in periodontal regeneration, both histologically and clinically [14,15,16]. However, some limitations to the use of EMDs remain, such as their limited performance in large defects, the storage conditions, and their animal origin, which can be a barrier for some patients and may carry some risk of zoonotic disease [17]. In this respect, Gungormus et al. [18] introduced a synthetic 22-amino-acid-long amylogenic peptide referred to as amelogenin-derived peptide 5 (ADP-5). This N-terminal peptide was shown to facilitate cell-free formation of a cementum-like hydroxyapatite mineral layer on demineralized human root dentin. According to Amin et al. [19], synthetic peptides could have an important potential for periodontal tissue repair and regeneration. The authors revealed the mechanisms mediating the interactions of peptide and periodontal ligament cells in vitro, such as binding to cell membranes, trans-Golgi network, and lysosomal vesicles. The same authors underlined the advantage of using synthetic peptides, which are animal-free origin, more cost effective, and their chemical synthesis could be more controlled according the targeted clinical application. Furthermore, various in vitro and preclinical studies have shown the beneficial effect of synthetic amelogenin peptides in the remineralization of hard dental tissues [20,21,22].

The purpose of the present study is to assess the in vitro effect of the ADP-5 N-terminal peptide, alone and included in an experimental gellan–xanthan hydrogel (GX), on periodontal cell behavior. Two hypotheses were suggested: (1) ADP-5 would enhance periodontal cell behavior (proliferation, mineralization ability, and inflammatory profile) when it is tested in direct contact alone and (2) the incorporation of ADP-5 into an experimental hydrogel would not affect its biological activity.

## 2. Materials and Methods

### 2.1. ADP-5 Synthesis

The ADP-5 was synthetized at the Institut des Biomolécules Max Mousseron (SynBio3 platform, UMR CNRS 5247), using a sequence of 22 amino acids (PGYINLSYEKSHSQAINTDRTA). Briefly, the peptide chain was elongated by standard Fmoc solid-phase chemistry with a Liberty BlueTM Microwave Peptide Synthesizer (CEM Corporation, Matthews, NC, USA). Synthesis was conducted on a 0.1 mmol scale on an Fmoc-Ala-Wang resin. Deprotections were performed with a 20% piperidine in Dimethylformamide (DMF, 7 mL) solution. All coupling reactions were performed with 5 equiv of HATU (1-[Bis(dimethylamino)methylene]-1H-1,2,3-triazolo[4,5-b]pyridinium 3-oxide hex-afluorophosphate) in DMF (0.5 M, 1 mL), 5 equiv of amino acids in DMF (0.2 M, 2.5 mL), and 10 equiv of DIEA (N,N-Diisopropylethylamine) in DMF solution (2 M, 0.5 mL). Each deprotection and coupling reaction was performed at room temperature under nitrogen bubbling. Each cycle was characterized by two deprotection steps for 10 min, three washings of 5 mL DMF, followed by a double 25 min coupling step. After the assembly was complete, the peptide–resin was washed with CH_2_Cl_2_ and cleaved from the resin with a TFA/H_2_O/TIS (TFA: trifluoroacetic acid and TIS: triisopropylsilane) solution (95:2.5:2.5 *v/v/v*). The crude peptide was purified by reverse-phase liquid chromatography using a PLC2050 Armen-Gilson purification system on a Luna 5 µm C18 100 Å Axia 100 × 21.2 mm, column. The mass and purity (isolated mass = 5.3 mg, purity = 92% (HPLC); [M+3H]^3+^ = 822.7) of the peptide were checked on an Agilent 1260 UHPLC system, coupled to an SQ spectrometer (electrospray ionization mode, ESI+).

The scanning electron microscopy (SEM) analysis was performed to visualize the ADP-5 structure. The powder of peptide was sputter coated with copper (10 ng) and observed under SEM (SEM, FEI-Quanta 250—Thermo Fisher Scientific, Illkirch-Graffenstaden, France).

### 2.2. Incorporation of the ADP-5 Peptide in the GX Hydrogel

GX powder was formed from a mixture of Gellan (P8169, Phytagel™, Sigma Aldrich, Saint-Quentin-Fallavier, France) and Xanthan powders (G1253, Sigma Aldrich, France) at a ratio of 9:1 wt%. All the hydrogels were prepared from this mixture with cold PBS: Phosphate-Buffered Saline (14190144: Thermo Fisher Scientific, Illkirch-Graffenstaden, France) buffer solution; a 30 s vibration using the vibrator Softly Satelec (Henry Schein, Limonest, France) was then applied to enable the hydrogel crosslinking. Several gel densities were tested and the GX hydrogel at the density of 3% was selected to be used in this study due to its mechanical properties and comparable clinical handling to the commercial Emdogain^®^ (EMD: Straumann, Basel, Switzerland). The ADP-5 peptide was incorporated (mixed with the GX powders directly) in the selected GX hydrogel at the concentrations of 100 and 200 µg/mL. The GX/ADP-5 mixture was first prepared using PBS, and then immerged in the culture media before the cell contact.

The list of tested samples used in the first and the second investigation steps, including synthetic peptide, and experimental and commercial gels, are shown in Table 1. The first investigation refers to the assessment of ADP-5 peptide alone and the second investigation refers to the assessment of ADP-5 factionalized experimental GX hydrogel.

### 2.3. Cell Culture

Human gingival fibroblasts (HGF) were isolated and harvested from healthy gingival tissue biopsies of patients, taken during routine orthodontic extractions. The collection of human dental tissue was conducted in compliance with French legislation at the University of Lyon 1—Hospices civils of Lyon, France. Human periodontal ligament cells (hPDL) were derived from human primary cell culture (#2630, ScienCell, San Diego, USA). Osteoblasts human cell line MG63 was obtained from American Type Culture Collection (ATCC^®^ CRL-1427™). Cementoblasts were derived from a mouse cell line (line OCM.30 mouse cementoblasts, ABM—Applied Biological Materials Inc, Richmond, USA.). HGF, MG63, and OCM.30 were cultured in Dulbecco’s Modified Eagle’s Medium (11995065: Thermo Fisher Scientific, Illkirch-Graffenstaden, France) supplemented with 10% fetal bovine serum (#0500, ScienCell, San Diego, USA), 1% penicillin/streptomycin (#0513, ScienCell, USA), and 0.2% amphotericin B (#0523, ScienCell, USA). hPDL were cultured in fibroblast medium (#2301, ScienCell, USA) supplemented with 10% fetal bovine serum, 1% penicillin/streptomycin, and 1% fibroblast growth supplement (#2352, ScienCell, USA). Cells were maintained at 37 °C under a humidified atmosphere of 5% CO2 in air. On achieving confluence, the cells were subcultured and passaged after a rinse with Dulbecco’s Phosphate Buffered Saline 1× Modified (D8537, Gibco™, France), taken down with 2.5% trypsin (15090, Gibco™, France) for 3 min. Then, cells were pelleted after 5 min of centrifugation at 1200 rpm and they were resuspended with cell medium and kept in culture flasks. The media associated with each cell type were changed every 2 days, and cells were passaged every 5 days. Cell cultures were examined routinely under an inverted microscope (CKX41, Olympus, Rungis, France).

### 2.4. Cell Metabolic Activity

Cell metabolic activity was evaluated using the Alamar blue assay (DAL1100, Thermo Fisher Scientific, France), which is colored blue in its oxidized state and becomes pink when reduced to the resorufin by metabolically active cells. Briefly, cells were seeded on 24-well culture plates at 104 cells/mL, according to the international organization for standardization (ISO) 10993-norm recommendations [23]. They were cultured in a regular medium, with several conditions in two experimental conditions. In the first part, the four periodontal cells (HGF, hPDL, MG63, and OCM.30) were cultured in direct contact with the synthetic ADP-5 at the following concentrations: 5, 10, 50, 100, and 200 µg/mL. In the second part of the experimental protocol, the same cell types were cultured in the presence of either GX hydrogel, or GX hydrogel with 100 µg/mL (GX-100) or 200 µg/mL (GX-200) ADP-5 peptide, or 100 µg/mL or 200 µg/mL commercial gel Emdogain^®^ (diluted in the culture media) (Emd^®^100 and Emd^®^200, respectively) (Table 1). After 1, 3, and 7 days of contact, Alamar blue solution was added directly into wells at the final concentration of 10% *v*/*v* and plates were incubated at 37 °C for 7 h. The amount of resorufin formed was determined by measuring absorbance intensity (570/600 nm), using an absorbance plate reader Infinite (Nanoquant infinite M200 pro, Tecan group Ltd., France). The results were expressed through the percentage of viability amount to 100% of viability of the control cells (cells without peptide for the first investigation step and cells without peptide or gel for the second investigation step).

### 2.5. Cell Morphology

The cell morphology was observed using confocal microscopy to evaluate the incidence of 7 days of direct contact with the tested peptide at the 50 and 100 μg/mL concentration (for hPDL and MG63). Fluorescence staining was performed to observe the formation and the organization of stressed fibers and morphological changes. Cells were washed three times with PBS. Then, they were fixed for 30 min by incubating in 3.7% formaldehyde (10426730, Thermo Fisher Scientific, France) in PBS, followed by further washing. The cells were permeabilized with 1% triton ×100 (×100, Sigma Aldrich, Saint-Quentin-Fallavier, France) in PBS, and then blocked with 1% Corning bovine serum albumin (354331: Thermo Fisher Scientific, Illkirch-Graffenstaden, France) in PBS. Actin microfilaments were stained by Alexa Fluor™ 488 Phalloidin (A12379, Thermo Fisher Scientific, France) at a 1:100 ratio at room temperature to visualize cell actin filaments (green fluorescence). Cell nuclei were identified using propidium iodide (P3566, Thermo Fisher Scientific, France) at a 1:3000 ratio at room temperature (red fluorescence) to visualize nuclei. Supercontinuum white-light laser was used to excite Alexa Fluor^®^488 and propidium iodide. Acquisitions were collected sequentially (green fluorescence/red fluorescence) to avoid potential crosstalk between the two channels. The resulting stained cells, in 1% bovine serum albumin in PBS, were examined under a confocal laser scanning microscope (CLSM LEICA SP5X Leica, Wetzlar, Germany).

### 2.6. Mineralization Ability of hPDL and OCM.30 Cells

Alizarin red staining (ARS) was used to assess matrix mineralization. In the first step of the investigation, the ARS assay was carried out following the contact of ADP-5 alone, at the concentration of 100 µg/mL and 200 µg/mL, after 7 and 14 days for both hPDL and OCM.30 cells. In the second part of the investigation, hPDL cells were subjected to 100 µg/mL and 200 µg/mL GX-ADP-5 or Emdogain^®^ (100 µg/mL and 200 µg/mL). The culture media were changed every 3 to 4 days.

For the ARS analysis, cells were firstly fixed using formaldehyde (3.7% in PBS, 30 min) and washed with deionized water. Then, 40 mM of ARS solution (pH 4.2) was added into the 24-well plates. The cells were incubated at room temperature for 40 min, then washed 3 times with deionized water and viewed under an optical microscope (CKX41, Olympus, France). For quantitative calcium analysis (semi-quantification) of mineralized matrix nodules generated from hPDL and OCM.30, the cells were treated with 10% cetylpyridinium chloride solution (C0732: Sigma-Aldrich, Saint-Quentin-Fallavier, France) for 30 min at room temperature to dissolve and release the calcium-combined ARS. The optic density values were read at 560 nm, which represented the relative quantity of deposited calcium in the mineralization nodules. The experiments were repeated at least 3 times (*n* = 9).

### 2.7. Inflammatory Mediators Production

ELISA assays were used on hPDL and OCM.30 cells to quantify the production of three inflammatory mediators: a growth factor, vascular endothelial growth factor A (VEGF-A), and two inflammatory cytokines, tumor necrosis factor alpha (TNF-α) and interleukin 11 (IL-11), involved in inflammatory reaction periodontitis [24,25]. These assays were made at 3 days following direct contact of each two-cell types with GX, GX-100, GX-200, Emd^®^100, and Emd^®^200. All cell lysates were subjected to ELISA for inflammatory mediators by applying ELISA kits: human (for hPDL) and mouse (for OCM.30) VEGF-A (KHG011, ThermoFisher Scientific), TNF-α (BMS223HS, ThermoFisher Scientific, France), and IL-11 (human EHIL11 and mouse EMIL11, ThermoFisher Scientific, France). Each ELISA was performed according to the manufacturer’s instruction. The experiment was performed in triplicate and repeated three times (*n* = 9); the data were compared to a standard curve. Absorption measurements were performed at 450 nm (Nanoquant infinite M200 pro, Tecan group Ltd., Lyon, France).

### 2.8. Statistical Analyses

Statistical analyses were performed by using one-way analysis of variance (ANOVA). An initial comparison was made between the control group (cells without any treatment) and the tested groups (cells in contact with ADP-5 alone or within the experimental gel versus Emdogain^®^). Then, the Mann–Whitney test and the Wilcoxon signed rank tests were used for comparison between the different investigated groups. The level of significance was set at * *p* < 0.05 and ** *p* < 0.001.

## 3. Results

### 3.1. ADP-5 Structure by SEM

**Figure 1:**
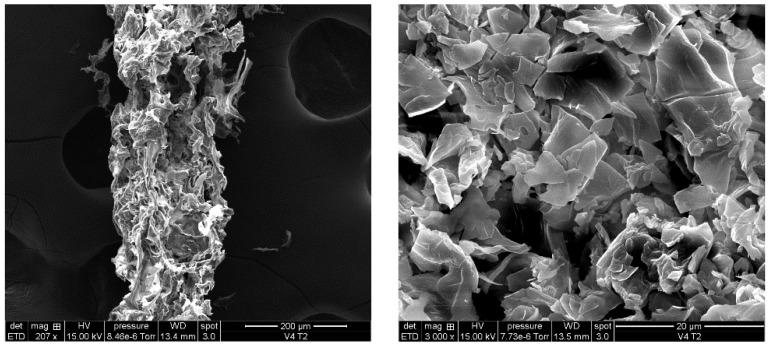
Morphological characterization of ADP-5 powder by SEM.

SEM analysis (Figure 1) demonstrates the morphology of ADP-5 peptide. Regular surface morphology of the peptide can be shown.

### 3.2. Cell Metabolic Activity

#### 3.2.1. Effect of ADP-5 Alone on Cell Viability

**Figure 2:**
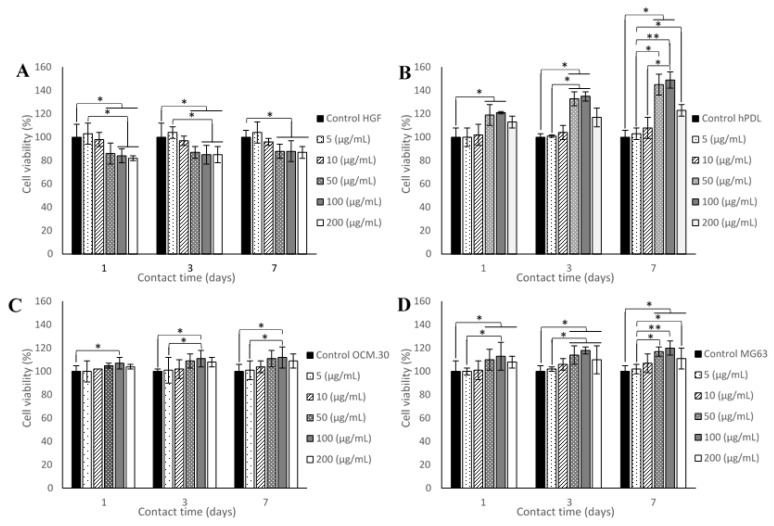
Cell metabolic activity expressed as viability rate (%) as function of cell type, ADP-5 concentrations, and contact time: (**A**) human gingival fibroblast (HGF) cells; (**B**) human periodontal ligament (hPDL) cells; (**C**) cementoblast (OCM.30) cells; and osteoblast (MG63) cells (**D**); data are mean ± SD of 3 independent experiments (*n* = 9). * *p* < 0.05 and ** *p* < 0.001 denote significant differences.

The results of cell metabolic activity, comparing the different concentrations of ADP-5 on the four periodontal cell types, are shown in Figure 2. A slight and significant decrease in cell viability was observed for HGF cells from the concentration of 50 μg/mL, whatever the contact time point (1, 3, and 7 days), compared to control cells. For the other three cell types (hPDL, OCM.30, and MG63), a cell viability significant enhancement was observed from day 1 of incubation with 50 μg/mL ADP-5 for MG63 and hPDL and 100 μg/mL ADP-5 for OCM.30 cells. hPDL cell viability revealed a significant enhancement at the concentrations of 50 μg/mL and 100 μg/mL; for OCM.30, a significant enhancement in cell viability was only observed at 100 μg/mL ADP-5; for MG63, the three concentrations (50 μg/mL, 100 μg/mL, and 200 μg/mL ADP-5) induced a significant enhancement in cell viability versus control cells.

#### 3.2.2. Effect of GX Hydrogels Containing ADP-5 Versus Emdogain^®^ Gels on Cell Viability

**Figure 3:**
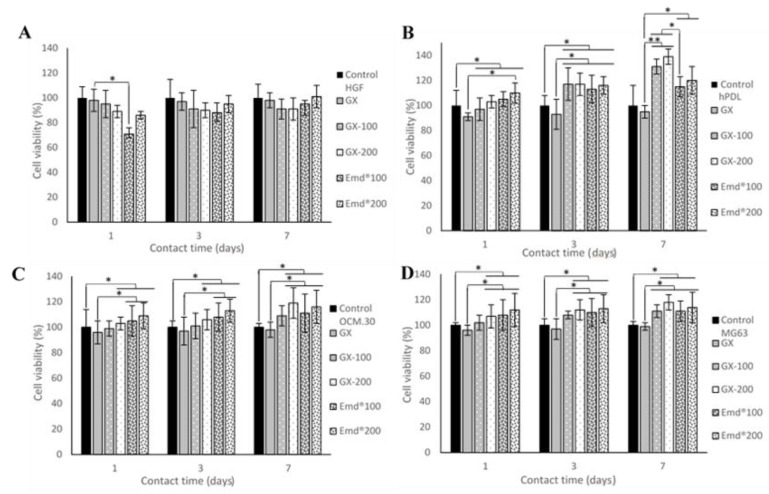
Cell metabolic activity expressed as viability rate (%) as function of the tested gel groups and the contact time: (**A**) human gingival fibroblast (HGF) cells; (**B**) human periodontal ligament (hPDL) cells; (**C**) cementoblast (OCM.30); (**D**) osteoblast (MG63) cells. Data are mean ± SD of 3 independent experiments (*n* = 9/group). * *p* < 0.05 and ** *p* < 0.001 denote significant differences.

The cell metabolic activity demonstrated no cytotoxic effect whatever the gel treatment conditions (Figure 3). For HGF cells, no statically significant difference was observed among all the tested gel groups. The other cell types exhibited a significant increase in cell metabolic activity in the presence of both GX containing ADP-5 and EMD regardless of the contact time. The most stimulation was found for hPDL following 7 days of contact with both ADP-5 and EMD. This enhancement occurred since the first day of contact at the concentration of 100 μg/mL. For the tested OCM.30 cementoblasts and MG63 osteoblasts cells, a significant increase was observed beginning at the concentration of 100 μg/mL of Emdogain and at 200 μg/mL of GX-ADP-5. It seems that the incorporation of ADP-5 in the GX hydrogel did not affect its active effect regarding the tested four periodontal cells.

### 3.3. Cell Morphology and Spreading

**Figure 4:**
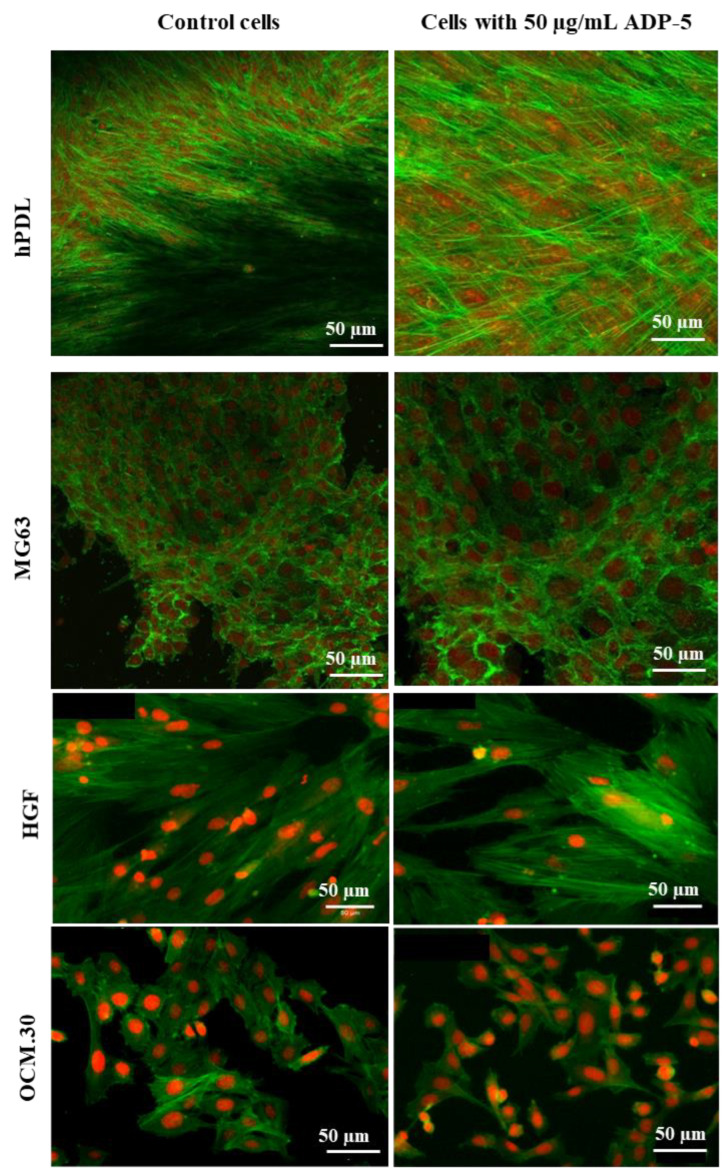
Representative images of hPDL, MG63, HGF, and OCM.30 cells’ morphology by confocal microscopy: control cells; cells in contact of 50 µg/mL of ADP-5. Cytoskeletal F-actin is stained green with Alexa Fluor™ 488 Phalloidin and cell nuclei are stained red with propidium iodide. Scale bar = 50 µm.

Figure 4 shows cell spreading of the four tested cell types after 50 µg/mL of ADP-5 contact. More abundant hPDL cells were observed in the presence of ADP-5 peptide than in the control cells without the peptide. hPDL cells predominantly exhibited a well-organized cytoskeletal architecture, as seen in actin cytoskeleton staining. Good cell adhesion and spreading in contact with the tested ADP-5 peptide was also observed. Similar observations to hPDL cells could be seen for the osteoblast MG63 cells. For the HGF, in the presence of 50 µg/mL of ADP-5, the nucleus was less abundant, unlike the OCM.30, which exhibited more nucleus (more cells) in the presence of 50 µg/mL of ADP-5 (Figure 4).

### 3.4. hPDL and OCM.30 Mineralization Ability by Alizarin Red Staining

**Figure 5:**
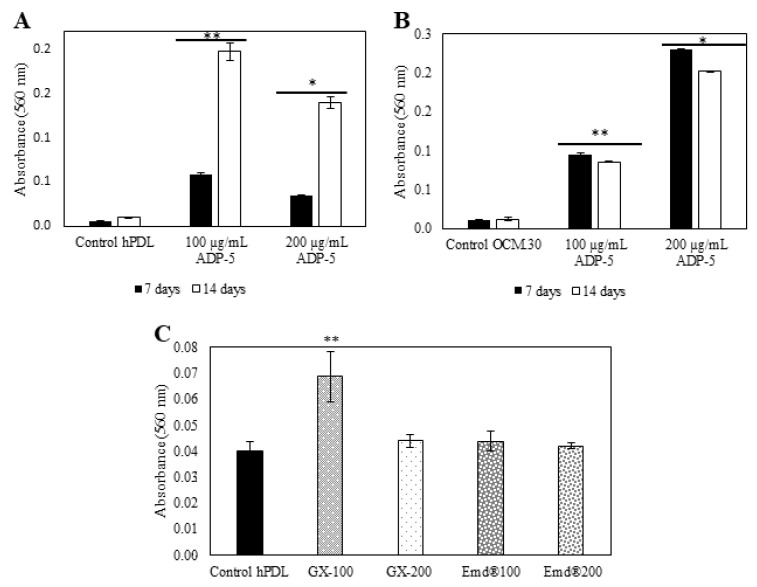
Calcium deposition quantification by Alizarin red staining of hPDL (**A**) and OCM.30 (**B**) cells as a function of contact time and peptide concentration. Data are mean ± SD of 3 independent experiments (*n* = 6). * *p* < 0.05 and ** *p* < 0.001 denote a significant difference. Calcium deposition quantification by Alizarin red staining of hPDL (**C**) cells following hydrogel contact groups (GX containing or not ADP-5 peptide or EMD). Data are mean ± SD of 3 independent experiments (*n* = 9/group). ** *p* < 0.001 denotes a significant difference (GX100 group compared to all the tested groups).

**Figure 6:**
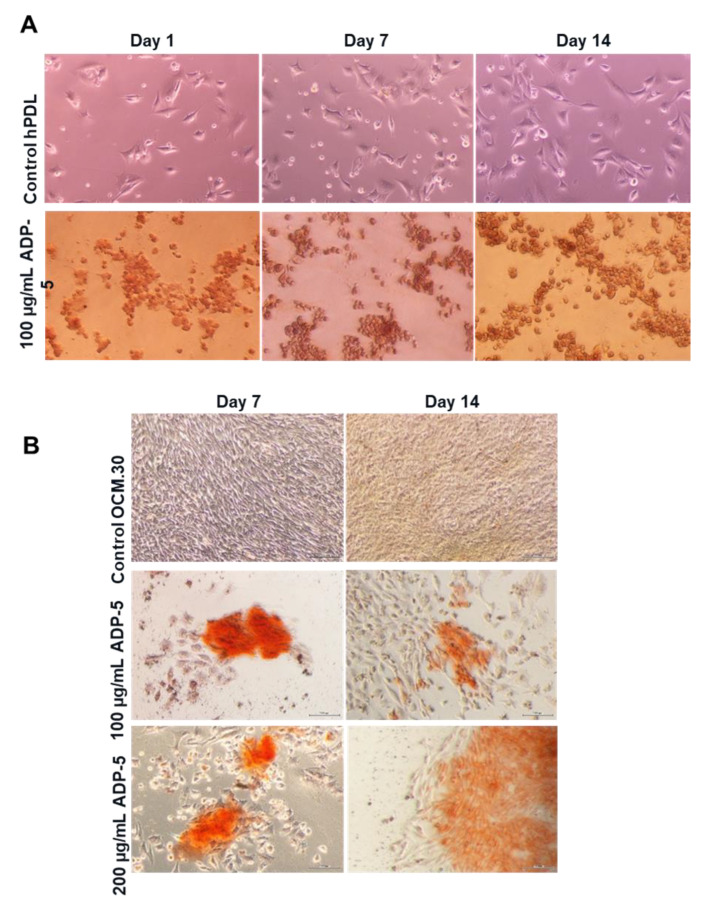
Representative optical images of Alizarin red staining: (**A**) hPDL and (**B**) OCM.30 cells as a function of contact time and peptide concentrations.

Figure 5A reveals an important enhancement of the extracellular calcium deposition amount in a time-dependent manner (from 7 to 14 days) in the hPDL treated with 100 µg/mL ADP-5 compared to the untreated hPDL cells. Interestingly, the lowest concentration (100 µg/mL) was more efficient than the highest concentration (200 µg/mL). More mineralization nodules were observed when hPDL cells were interfaced by ADP-5 at the concentration of 100 µg/mL, whatever the time point tested (Figure 6A). Regarding OCM cells, the Alizarin red quantification (Figure 5B) demonstrates a significant enhancement in calcium deposition, which is higher in the presence of 200 µg/mL of ADP-5 in a time-dependent manner. At the concentration of 100 µg/mL, a slight but significant increase was also shown with a time-dependent effect. When cells were interfaced by gel groups, the experimental functionalized group GX100 was the most efficient and revealed significant enhancement of the extracellular calcium deposition amount compared to all the tested gel groups (Figure 5C). Qualitatively, optical images of Alizarin red OCM.30 stained cells (Figure 6B) illustrate an evident increase in the mineralized nodules in the ADP-5 groups compared to the control groups (cells without ADP-5).

### 3.5. hPDL and OCM.30 Cells Inflammatory Mediator Quantification by ELISA Assay

**Figure 7:**
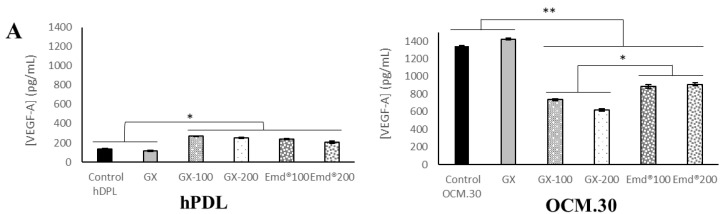

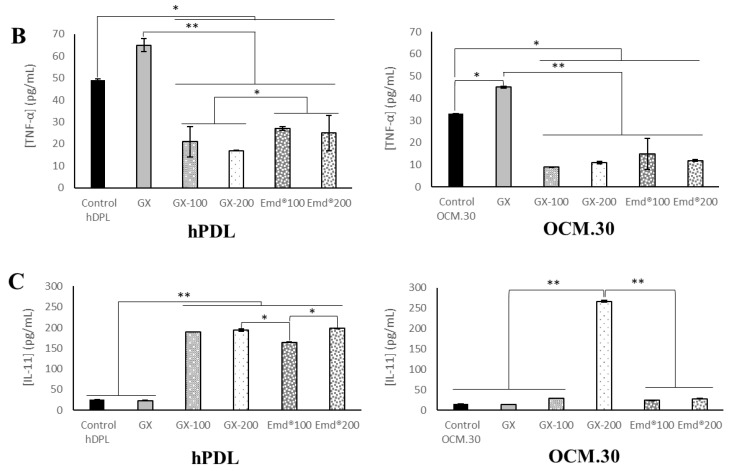
Rate of inflammation mediators production by ELISA after 3 days of cells’ contact with GX containing or not ADP-5 and EMD: protein rate of VEGF-A factor (**A**), TNF-α (**B**), and IL-11 (**C**) by hPDL (left side) and OCM.30 (right side) cells. Data are mean value of 3 independent experiments (*n* = 12/group). * *p* < 0.05 and ** *p* < 0.001 denote a significant difference.

The inflammatory profile of the two investigated periodontal cells (periodontal ligament cells and cementoblasts) was assessed by quantifying the protein level of three inflammatory factors: VEGF-A, TNF-α, and IL-11. The obtained data are presented in Figure 7. For both tested cell types, both GX hydrogel containing ADP-5 and Emdogain^®^ increased significantly both VEGF-A and IL-11, while the TNF-α protein amounts were decreased in contact with the two tested gels. Cytokine production remained unaffected when challenged by nonfunctionalized GX hydrogel. For OCM.30 cells, the production of IL-11 was enhanced only in the presence of 200 µg/mL GX–ADP-5.

## 4. Discussion

Periodontal tissue regeneration is the main aim of current periodontal therapies intended to recover damaged tissues. The periodontium complex structure, with alternating hard and soft tissues, makes its tissue regeneration a challenge. In this context, treatments, associated with periodontal wound healing and tissue regeneration have been extensively investigated [16,26,27,28]. Among the current periodontal regenerative therapy procedures, Emdogain^®^ is being widely used in clinical practice [14]. This gel of animal origin is mostly comprised of amelogenins, responsible for mineralization during tooth root formation, and which has inherent cementogenic properties [29,30]. On the other hand, Emdogain^®^, as well as guided tissue regeneration (GTR) and other therapies, can often be unpredictable for large defects [31,32,33,34]. Moreover, its animal origin remains a barrier for some patients. In this respect, recent research is directed towards synthetic peptide to overcome some of these limitations; therefore, the use of the ADP-5 synthetic peptide initially identified by Gungormus et al. [18] could provide a relevant answer to these patients and constitutes an alternative to animal sacrifice. ADP-5 peptide was reported to have a beneficial remineralization ability with a potential use for periodontal tissue regeneration. Nonetheless, the ADP-5, in its initial form of powder, needs to be incorporated in a carrier “transporter” to facilitate its use and preserve its active potential in periodontal pockets and in different clinical situations. Within this framework, the use of ADP-5 peptide-functionalized hydrogels as a modified bioactive scaffold may represent a relevant solution for periodontal complex regeneration, containing all of the required structural (such as easy handling and gel flexibility) and bioactive properties (such as ADP-5 mineralization ability) and lacking any immunogenic cellular components due to the absence of animal origin.

The concept of the current study was to compare and evaluate the in vitro biological behavior of an experimental hydrogel, based on gellan–xanthan mixture containing ADP-5 peptide. The in vitro behavior of the four representative periodontal cell types (gingival fibroblasts, osteoblasts, periodontal ligament cells, and cementoblasts) was first assessed in contact with ADP-5 alone in terms of cell proliferation, cell morphology, and cell remineralization. As a second investigation step, the effect of incorporating the tested ADP-5 into the experimental hydrogel scaffold, was then performed.

The assessment of experimental scaffold in contact with cells is a fundamental first step of biocompatibility and bioactivity evaluation. Cell biological testing should be conducted on specific cells according to the recommendations established by ISO 10993-5 [35]. The clinical applications have to be considered in order to select the suitable cell type regarding the targeted tissue with the aim to mimic as possible in vivo situations [36]. Periodontal ligament cells (hPDL) have been considered as the cell type with the highest potential for periodontal regeneration [37]. This could explain the large use of hPDL cells when assessing the performance of different periodontal therapies [38]. The effect on cell growth of EMD in a gel carrier versus EMD in a liquid carrier and their regulatory role of the inflammatory reaction was also investigated using PDL cells [39]. Moreover, cementoblast (OCM.30) cells were used for the same purpose, such as the potential use of plasminogen activation system as a specific target to control amelogenin-mediated tissue regeneration [40] and the potential autophagy effect of TNF-α and its critical role in cementoblasts differentiation, mineralization, and apoptosis [28,41,42].

The results of the present study indicate that ADP-5 peptide enhanced periodontal cells’ metabolic activity and cell spreading when assessed alone; this is in agreement with the results obtained by Gungormus et al. [18] regarding periodontal ligament cells. Moreover, the tested ADP-5 enhanced the behavior of three other investigated periodontal cell types, namely the osteoblasts, cementoblasts, and gingival fibroblasts. When the ADP-5 was incorporated into the experimental gellan–xanthan gel, it was found to behave identically to the commercial Emdogain^®^ regarding periodontal cells. Emdogain^®^ has previously demonstrated a unique capacity to promote periodontal cells’ mineralization in vitro [43]. The 100 µg/mL concentration induced the best cell response in the presence of the ADP-5 alone, and the 200 µg/mL was the highest concentration used that allowed the release of the active molecules from the experimental functionalized hydrogel. On the other hand, the obtained results demonstrated that this synthetic peptide stimulates the proliferation of cementoblasts, osteoblasts, and especially the periodontal ligament cells, while limiting the proliferation of gingival fibroblasts. This relative inhibition of gingival fibroblast proliferation could be of a major clinical relevance, since it prevents gingival tissue from interfering with periodontal attachment during the regeneration process [29,44]. To the best of our knowledge, this is the first study assessing four representative periodontal cells in contact with the ADP5 peptide. Hoang et al. [45] evaluated the effect of EMD on three cell types (PDL, HGF, and Mg63 cells) on wound healing using an in vitro wound model based on wounded cell monolayers in tissue culture plates. As in the current study regarding the ADP-5 peptide, the authors concluded that EMD stimulated periodontal ligament cell proliferation, which consequently improved periodontal regeneration. 

Since stimulation of cell proliferation alone does not accurately substantiate the biological behavior of cells during the regeneration process, mineralization ability, and the inflammatory profile of the two main representative periodontal cells (periodontal ligament as the representative of soft tissue and attach cells and cementoblasts as representative of hard tissue) were also assessed in the current study. Alizarin red S assay demonstrated more mineralization nodules and significant enhancement of the extracellular calcium deposition when cells were interfaced with both ADP-5 alone and ADP-5 functionalized experimental hydrogel compared to control cells. Alizarin red staining assay is commonly used to determine the mineralized nodule formation and quantify calcium deposits at the interface of cells–biomaterials, such as mesoporous bioactive glasses in contact with pulp cells [46], PDL cells after their stimulation with TNF-α [47], primary osteoblasts and PDL cells after contact with EMD [40], and MC3T3-E (mouse osteoblast cell line) when interfaced with strontium-doped bioactive glasses [48].

In the current study, the inflammatory profile was assessed using ELISA by quantifying the protein level to predict the inflammatory response induced by the ADP-5 peptide. The ELISA technique is widely used to quantify mediators [49,50,51,52,53]. Three key inflammatory mediators were investigated: a proinflammatory cytokine (TNF-α), an angiogenic growth factor (VEGF-A), and the anti-inflammatory cytokine (IL-11). It was previously reported that IL-11 is underexpressed in periodontitis [54,55], while TNF-α is overexpressed [55], there is a reduction of VEGF in periodontal disease [56], and these are correlated with the resolution of periodontal inflammation and periodontal tissue healing [57]. In the present study, both experimental GX hydrogel containing ADP-5 and Emdogain^®^ increased VEGF-A and IL-11 significantly and decreased TNF-α protein amounts. This is consistent with the finding of Almqvist et al. [58]; the authors demonstrated that monocyte-derived macrophages, stimulated by inflammatory agonist LPS, responded to the treatment with Emdogain^®^ by decreasing the TNF-α proinflammatory activity and enhancing the expression of tissue repair mediators such as VEGF. In a previous study, the production of inflammatory cytokine by monocytes was assessed using cDNA microarrays, and EMD downregulated the expression of genes involved in the early inflammatory phases of wound healing, such as interleukin (IL-6) [40]. Despite the dissimilarities with our study (cell type and methodology), these findings indicated that the decrease in proinflammatory markers (such as TNF-α) in response to the ADP-5 peptide could contribute to the downregulation of the proinflammatory response occurring after the placement of a scaffold during a regeneration process. Villa et al. [59] found that the proinflammatory cytokine secretion from human gingival fibroblasts decreased following treatment with EMD and its peptide fractions. In agreement with the current study, this funding underlined the biological activity of amelogenic peptides on proinflammatory cell behavior. 

In the current study, the GX experimental hydrogel was merely developed to provide a proof of concept for the ADP-5 carrier. Therefore, the main limitation of the study was that the experimental hydrogel used is different in composition from the commercial gel Emdogain^®^. Additional hydrogel compositions close to the one contained in Emdogain^®^ could also have been assessed. The characterization of the mechanical properties of the functionalized experimental hydrogel, as well as the kinetic release of ADP-5 from the GX hydrogel, should be assessed and controlled in the future; these could provide additional information regarding the functionalized scaffold potential clinical use.

From a clinical perspective, it will be necessary in future investigations to assess the experimental ADP-5 functionalized gellan–xanthan hydrogel in vivo, especially targeting periodontal regenerative procedures such as intraosseous or furcation lesion regeneration. The storage constraints of the commercial gel Emdogain^®^ represent a crucial point that may limit its use by dentists, especially those who are geographically isolated. The next step would be to investigate the storage constraints of the ADP-5 functionalized gellan–xanthan hydrogel suggested in the present study.

## 5. Conclusions

The present study highlights the superior in vitro biological behavior of the assessed synthetic amelogenin-derived peptide ADP-5, alone and within the resorbable scaffold, based on gellan–xanthan hydrogel. This behavior was revealed by:

- Enhanced proliferation, adhesion, and spreading of all periodontal cell types, and especially periodontal ligament cells;
- Slight inhibition of human gingival fibroblasts proliferation;
- Enhancement in periodontal cell mineralization potential;
- Improvement of key inflammatory mediators.

After further investigations, the elaborated bioactive scaffold-based ADP-5 peptide together with GX hydrogel could serve as a potential regenerative material for clinical practice. Moreover, the synthetic animal-free ADP-5 peptide could be used as a bioactive agent to functionalize different scaffolds for periodontal regeneration.

## Figures and Tables

**Table 1:** Summary of tested peptide and hydrogel samples.

**First investigation**		
**Cell types**	**Conditions (ADP-5 concentration (µg/mL))**	
**Human gingival fibroblasts (HGF)** **Human periodontal ligament (hPDL)** **Osteoblasts (MG63)** **Cementoblasts (OCM.30)**	Control cells (glass substrate, no ADP-5)	
	5	
	10	
	50	
	100	
	200	
**Second investigation**		
**Cell types**	**Groups**	**Conditions**
**Human gingival fibroblasts (HGF)** **Human periodontal ligament (hPDL)** **Osteoblasts (MG63)** **Cementoblasts (OCM.30)**	Control cells	Glass substrate, no hydrogel
	GX	3% GX hydrogel
	GX-100	3% GX hydrogel + 100 µg/mL ADP-5
	GX-200	3% GX hydrogel + 200 µg/mL ADP-5
	Emd^®^100	Emdogain^®^ 100 µg/mL (dilution compared to culture medium)
	Emd^®^200	Emdogain^®^ 200 µg/mL (dilution compared to culture medium)

## Data Availability

Not applicable.
